# Evaluation of Histidine–Octamer-Modified Hyaluronic Acid as a Cytosolic Drug Delivery Material via CD44-Mediated Cellular Uptake and Endosomal Escape

**DOI:** 10.3390/pharmaceutics18091137

**Published:** 2026-09-10

**Authors:** Tomona Yukimura, Hirono Ito, Takahiro Suzuki, Toshinobu Seki, Tomohiro Seki

**Affiliations:** 1 Faculty of Pharmacy and Pharmaceutical Sciences, Josai University, Saitama 350-0295, Japan; yy20019@josai.ac.jp (H.I.); sekt1042@josai.ac.jp (T.S.); 2 Department of Pharmacy, Saitama Medical University Hospital, Saitama 350-0295, Japan; bell.hawk@outlook.jp

**Keywords:** hyaluronic acid, histidine octamer, CD44 targeting, endosomal escape

## Abstract

**Background:** Although intracellular delivery via endocytosis is a promising strategy for drugs acting in the cytosol or nucleus, many macromolecular therapeutics remain trapped within endosomal/lysosomal compartments, limiting efficient cytosolic delivery. In this study, we designed a novel functional hyaluronic acid (HA)-based polymer, HA–octahistidine (His8), by conjugating His8 to CD44-targeting HA, and evaluated its physicochemical properties, cellular uptake, and endosomal escape capability. **Methods:** HA–His8 was synthesized via 1-ethyl-3-(3-dimethylaminopropyl)carbodiimide/N-hydroxysuccinimide (EDC/NHS)-mediated coupling and characterized using ^1^H NMR spectroscopy. Particle size and zeta potential were measured via dynamic light scattering, and buffering capacity was evaluated using acid–base titration. Cellular uptake and intracellular localization were investigated in CD44-high MDA-MB-231 and CD44-low MCF-7 cells via confocal laser scanning microscopy. Cytotoxicity was evaluated using the 3-(4,5-dimethylthiazol-2-yl)-2,5-diphenyltetrazolium bromide (MTT) assay. **Results:** Successful conjugation of His8 was confirmed with a degree of substitution of 4.66 mol% relative to HA carboxyl groups. HA–His8 exhibited a nanoscale hydrodynamic diameter under physiological conditions, which increased under acidic conditions together with changes in zeta potential. HA–His8 exhibited higher buffering capacity than free His8 and was preferentially internalized by CD44-high MDA-MB-231 cells compared with MCF-7 cells. Compared with unmodified HA, HA–His8 exhibited lower colocalization with LysoTracker that decreased over time, indicating reduced retention within acidic vesicles. HA–His8 also exhibited low cytotoxicity over the tested concentration range. **Conclusions:** His8 modification alters HA intracellular localization while preserving CD44 targeting, thereby facilitating endosomal escape. These findings highlight HA–His8 as a potential platform for the cytosolic delivery of macromolecular therapeutics, including proteins, peptides, and nucleic acids.

## 1. Introduction

Macromolecular biotherapeutics, including antibodies, proteins, and peptides, have attracted considerable attention as promising therapeutic modalities for cancer and a variety of other diseases [1,2]. Furthermore, advances in the development of macromolecular therapeutics, particularly molecularly targeted agents, have enabled treatment strategies with high specificity and multifunctionality that are difficult to achieve using conventional small-molecule drugs [3,4]. However, for macromolecular therapeutics that exert their effects intracellularly, their large molecular size prevents efficient passive diffusion across the plasma membrane, necessitating the use of alternative mechanisms for cellular entry. Endocytosis represents one promising strategy for intracellular drug delivery [5,6,7]. However, therapeutics internalized via this pathway are subsequently transported from endosomes to lysosomes, where drugs intended to act in the cytosol often fail to reach their site of action and may be degraded or inactivated by the acidic environment and hydrolytic enzymes, thereby limiting their intracellular therapeutic efficacy [8,9]. Therefore, the development of drug delivery systems (DDSs) that not only promote efficient cellular uptake but also facilitate endosomal escape into the cytosol has become an important challenge [10,11,12].

Various strategies have been investigated to overcome this endosomal barrier, including membrane-fusogenic peptides, pH-responsive polymers, and membrane-disruptive materials [13,14,15]. Among them, histidine-containing peptides have attracted considerable interest as endosomal escape-promoting materials [12,16]. The imidazole group of histidine (*pK_a_* ≈ 6.0) is protonated under the mildly acidic conditions of endosomes (pH 5.0–6.5), providing buffering capacity within this pH range [17]. This buffering effect has been proposed to promote osmotic swelling of endosomes through increased ion and water influx (the proton sponge effect), thereby facilitating the intracellular release of internalized cargo [18,19]. Additionally, the amphoteric nature of the imidazole group may enhance membrane interactions under acidic conditions, further promoting intracellular trafficking [12,16,20].

Hyaluronic acid (HA) has been widely investigated as a carrier material for tumor-targeted drug delivery because of its specific interaction with the CD44 receptor, which is overexpressed in several cancer cells [21,22,23]. HA is preferentially internalized through CD44-mediated endocytosis and possesses excellent biocompatibility, biodegradability, and low immunogenicity [24,25,26,27]. The carboxyl groups of HA play an essential role in CD44 recognition [28,29]. Nevertheless, even when HA efficiently delivers cargo into cells, the therapeutic efficacy remains limited if the delivered materials are retained within the endosomal or lysosomal compartments [30].

HA-based materials with endosomal escape functionality could potentially be applied to the cytosolic delivery of various therapeutic cargos, including macromolecular therapeutics such as proteins, peptides, and nucleic acids, as well as small-molecule therapeutics that require access to intracellular targets. Because these cargos differ in physicochemical properties such as molecular weight, charge, and hydrophobicity, appropriate loading strategies should be selected according to the characteristics of each cargo. Potential approaches include covalent conjugation through available functional groups of HA or additionally introduced reactive groups, attachment through cleavable linkers, and non-covalent loading based on electrostatic or hydrophobic interactions.

**Figure 1:**
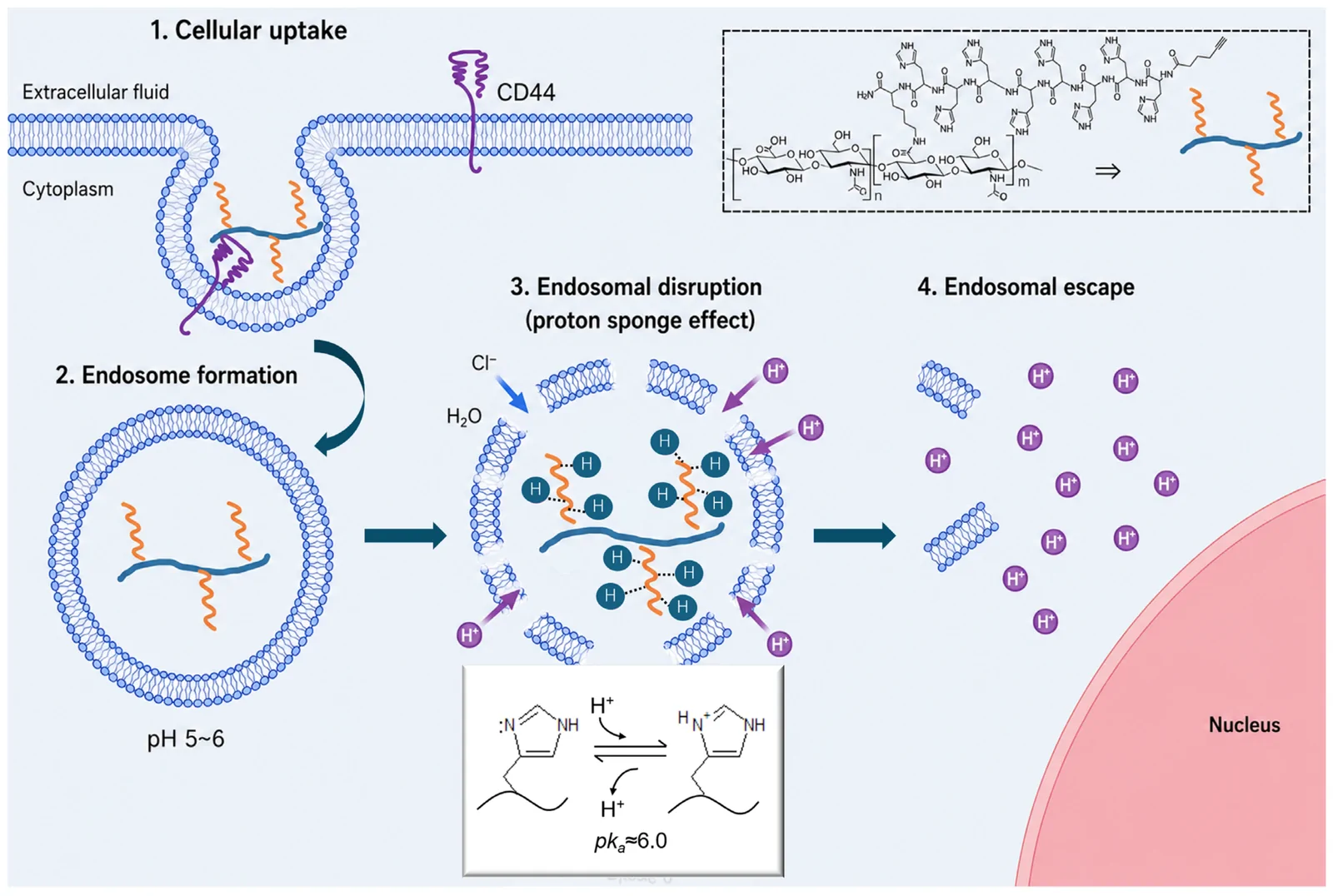
Schematic illustration of CD44-targeted intracellular delivery and enhanced endosomal escape of hyaluronic acid–octahistidine (HA–His8).

Based on this background, the development of intracellular delivery materials integrating CD44 targeting with endosomal escape functionality represents a promising strategy for constructing DDSs for therapeutic molecules requiring cytosolic delivery. In this study, HA with a molecular weight of approximately 45 kDa was selected to balance CD44 recognition and chemical modification, and octahistidine (His8) was introduced while preserving as many of the carboxyl groups involved in CD44 interactions as possible (Figure 1). Rather than developing a finalized DDS loaded with a specific therapeutic cargo, this study was designed as a proof-of-concept investigation to construct HA–His8 as a functional polymeric material for potential application in future intracellular delivery systems and to characterize its fundamental physicochemical properties and intracellular behavior. Specifically, we investigated whether His8 modification could impart buffering capacity under acidic endosomal conditions and alter intracellular localization while preserving the preferential cellular uptake of HA in CD44-high cells.

## 2. Materials and Methods

### 2.1. Materials

Potassium dihydrogen phosphate, disodium hydrogen phosphate, anhydrous magnesium sulfate (MgSO_4_), sodium chloride (NaCl), dichloromethane (DCM), hydrochloric acid (HCl), tetrahydrofuran (THF), N-dimethylformamide (DMF), acetonitrile (MeCN), ethyl acetate (EtOAc), dimethyl sulfoxide (DMSO), chloroform, piperidine and trifluoroacetic acid (TFA) were purchased from Fujifilm Wako Pure Chemical Industries (Osaka, Japan). N,N-Diisopropylethylamine (DIPEA), Triisopropylsilane (TIPS), 1-ethyl-3-(3-dimethylaminopropyl)carbodiimide (EDC·HCl), and tetrabutylammonium hydroxide (TBAOH) were purchased from Tokyo Chemical Industry Co., Ltd. (Tokyo, Japan). Rink Amide resin and Fmoc-Lys (Mtt)-OH were purchased from Watanabe Chemical Industry Ltd. (Hiroshima, Japan). Sulfo-Cyanine5.5 amine (Cy5.5) was purchased from Lumiprobe (Hunt Valley, MD, USA). Fmoc-His(Trt) and ethyl 2-cyano-2 ((dimethyliminio)(morpholino)methyloxyimino) acetate hexafluorophosphate (COMU) were purchased from the Peptide Institute (Osaka, Japan). α-Cyano-4-hydroxycinnamic acid and 4-kDa fluorescein isothiocyanate (FITC)–dextran (FD-4) were purchased from Sigma-Aldrich (St. Louis, MO, USA). Potassium chloride (KCl) was purchased from Kanto Chemical Co., Ltd. (Tokyo, Japan). The LysoTracker Green DND-26 was purchased from Thermo Fisher Scientific (Waltham, MA, USA).

### 2.2. Cells

Human breast-cancer-derived MDA-MB-231-Luc (MDA-MB-231) (JCRB1559) and MCF-7-Luc (MCF-7) (JCRB1372) cells were obtained from the Human Science Resource Bank (Osaka, Japan). Cells were cultured in Dulbecco’s modified Eagle’s medium supplemented with 10% fetal bovine serum and 1% penicillin–streptomycin solution at 37 °C in a humidified atmosphere containing 5% CO_2_.

### 2.3. Peptide Synthesis and Purification

An N-terminal alkyne-functionalized octahistidine peptide with a C-terminal lysine residue (Alk-His-His-His-His-His-His-His-His-Lys-NH_2_, His8) was synthesized by standard 9-fluorenylmethoxycarbonyl (Fmoc) solid-phase peptide synthesis [31]. The N-terminal alkyne group was incorporated as a reactive handle for future conjugation of drugs or other functional molecules via click chemistry. Rink amide resin (0.2 mmol g^−1^ loading) was swollen in DMF for 30 min in a fritted syringe reactor (HENKE, HJ4050-LT) with gentle agitation. The Fmoc protecting groups were removed by treatment with 20% piperidine in DMF (3 min, followed by 12 min), and the resin was washed with DMF (×3). Amino acid coupling was performed using Fmoc-protected amino acids (4 equiv), COMU (4 equiv), and DIPEA (8 equiv) in DMF (0.2 M) for 1 h under agitation. The peptide sequence was assembled from the C-terminus to the N-terminus of the resin. After completion of the peptide chain elongation and removal of the terminal Fmoc group, the N-terminus of the resin-bound His_8_-Lys peptide was coupled to an alkyne-containing carboxylic acid (4 equiv), COMU (4 equiv), and DIPEA (8 equiv) in DMF (0.2 M) for 3 h under agitation to obtain the N-alkyne-functionalized peptide on the resin.

The peptide was cleaved from the resin using TFA: TIPS: water ratio of 95:2.5:2.5 (*v*/*v*/*v*) for 3 h at room temperature. The cleavage solution was filtered, and the TFA was removed under reduced pressure. The crude peptide was dissolved in acetonitrile/water (3:7, *v*/*v*) containing 0.1% TFA and purified by reversed-phase flash chromatography (Biotage Isolera Prime) using a gradient of acetonitrile and water containing 0.1% TFA.

Fractions containing the target peptides were identified by Matrix-assisted laser desorption/ionization time-of-flight mass spectrometry (MALDI-TOF-MS) (JEOL JMS-S3000). The matrix solution consisted of α-cyano-4-hydroxycinnamic acid (10 mg mL^−1^) in acetonitrile/0.1% TFA aqueous solution (1:1, *v*/*v*). The eluent (1 μL) was mixed with the matrix (10 μL), spotted onto a target plate, and air-dried prior to analysis. Spectra were acquired in the positive ion mode with a spiral ion trajectory and an acceleration voltage of 20 kV. External calibration was performed using Poly(ethylene glycol) (PEG) 800–1500 ([M + Na]^+^) (MALDI-TOF-MS: m/z calcd for [M + H]^+^, 1336.4283; found, 1336.6437). The combined fractions were lyophilized to obtain His8.

### 2.4. Preparation of HA–TBA

HA (5.0 g) was dissolved in distilled water (830 mL) at room temperature. The cation exchange resin (45 g) was washed three times with distilled water and added to the HA solution. The suspension was stirred for 3 h at room temperature. The resin was removed by filtration through a glass filter, and the filtrate was neutralized to pH 8.0 using TBAOH. The resulting solution was lyophilized to obtain HA–TBA as a white powder [32].

### 2.5. Synthesis of HA–His8

HA–TBA (80.4 mg, 1.0 μmol; 112 μmol of carboxyl groups) was dissolved in anhydrous DMSO (3.5 mL). EDC·HCl (85.88 mg, 448 μmol) and NHS (51.52 mg, 448 μmol) were added, and the reaction mixture was stirred for 1 h at room temperature to activate the carboxyl groups.

His8 (74.84 mg, 56 μmol) dissolved in anhydrous DMSO (5.0 mL) was added dropwise to the activated HA solution, followed by DIPEA (52.4 μL, 308 μmol). The reaction mixture was then stirred at room temperature for 24 h.

The reaction mixture was dialyzed against excess distilled water for 24 h, during which time the external water was replaced 2–3 times. The product was then dissolved in 5% NaCl solution at an HA concentration of 10 mg/mL and dialyzed against distilled water for 24 h using regenerated cellulose dialysis tubing (MWCO 15 kDa). The purified solution was then lyophilized to obtain HA–His8 as a white powder [33].

### 2.6. Synthesis of Cy–HA

See Supporting Information for details.

### 2.7. Synthesis of Cy5.5-Labeled HA–His8 (Cy–HA–His8)

See Supporting Information for details.

### 2.8. Characterization of HA–His8

The lyophilized powder (2.0 mg) was dissolved in phosphate-buffered saline (PBS, 1.0 mL, pH 7.4) or acetate buffer (1.0 mL, pH 5.0 or pH 4.0) adjusted to the same ionic strength as PBS (I = 0.166 M). The hydrodynamic diameter, zeta potential, and polydispersity index (PDI) were measured via dynamic light scattering (DLS) using a Zetasizer Nano ZS (Malvern Panalytical Ltd., Malvern, UK). Measurements were performed in triplicate at room temperature.

### 2.9. Evaluation of the Buffering Capacity of HA–His8

The buffering capacity of HA–His8 was evaluated by acid–base titration. HA and HA–His8 were dissolved in 0.01 M NaOH containing 100 mM NaCl at a concentration of 3.0 mg/mL. His8 was dissolved in the same solution (0.47 mg/mL) to adjust its molar concentration to that of the HA–His8 solution. The sample solution (10 mL) was first adjusted to pH 11.0 with 1 M NaOH. Subsequently, 0.01 M HCl was added in 0.063 mL increments at 30 s intervals until the pH reached 3.0. The pH was recorded after each addition, and titration curves were plotted as a function of the added HCl volume.

The buffering capacity was calculated as the percentage of protonatable amino groups over the pH range of 7.4–4.0 using the following equation:

$$\mathrm{Buffering}\;\mathrm{capacity}\;\left(\%\right)=\frac{\left(\Delta V_{HCl}-\Delta V_{Blank}\right)C_{HCl}}{N\;mol}\times 100$$

where $V_{\mathrm{sample}}$ and $V_{\mathrm{blank}}$ are the volumes of 0.01 M HCl consumed by the sample and blank, respectively, between pH 7.4 and 4.0, and N is the total molar amount of protonatable amino groups in the sample.

### 2.10. Intracellular Localization and Endosomal Escape of HA–His8

Cells (4 × 10^4^ cells/well) were seeded onto poly-L-lysine-coated glass coverslips (Matsunami Glass Co., Ltd., Osaka, Japan), placed in 12-well plates, and incubated overnight at 37 °C in a humidified atmosphere containing 5% CO_2_. After washing with PBS, Cy–HA–His8 (0.5 mg/mL) was added and the cells were incubated for the indicated period. One hour before the end of the incubation, LysoTracker Green DND-26 (10 µM) was added. The cells were then washed three times with PBS, fixed with 4% paraformaldehyde for 15 min, and mounted using the VECTASHIELD mounting medium containing 4′,6-diamidino-2-phenylindole(DAPI). Fluorescent images were acquired using an FV1000-D confocal laser scanning microscope (Olympus Corporation, Tokyo, Japan). Colocalization between Cy–HA–His8 and LysoTracker Green was quantified by calculating the Pearson’s correlation coefficient using ImageJ software 1.54p (National Institutes of Health, Bethesda, MD, USA) with the Coloc 2 plugin. Line profile analysis was performed using the ImageJ software 1.54p to evaluate the fluorescence intensity distribution across representative cells.

### 2.11. Endosomal/Lysosomal Leakage Assay Using FD-4

Cells (4 × 10^4^ cells/well) were seeded onto poly-L-lysine-coated glass coverslips (Matsunami Glass Co., Ltd., Osaka, Japan), placed in 12-well plates, and incubated overnight at 37 °C in a humidified atmosphere containing 5% CO_2_. The cells were then incubated with 4-kDa FITC–dextran (FD4, 2 mg/mL) alone or in combination with Cy–HA or Cy–HA–His8 (0.5 mg/mL) for 6 h. After incubation, the cells were washed three times with PBS, fixed with 4% paraformaldehyde for 15 min, and mounted using VECTASHIELD mounting medium containing DAPI. Fluorescence images were acquired using an FV1000-D confocal laser scanning microscope (Olympus Corporation, Tokyo, Japan) under identical acquisition settings for all treatment groups.

Intracellular FD4 fluorescence intensity was quantified using ImageJ software (National Institutes of Health, Bethesda, MD, USA). For assessment of the intracellular FD4 distribution, cells were classified as exhibiting either a punctate or diffuse fluorescence pattern. Cells showing FD4 fluorescence predominantly confined to discrete intracellular puncta were classified as punctate, whereas cells showing FD4 fluorescence that was not restricted to discrete puncta but was distributed continuously over a broader intracellular area were classified as diffuse-positive. The same image display settings were applied to all treatment groups during classification. The percentage of diffuse-positive cells was calculated as the number of diffuse-positive cells divided by the total number of analyzed cells × 100. Cells pooled from three microscopic fields were evaluated for each treatment group.

### 2.12. Evaluation of Cytotoxicity of HA–His8

Cell viability was evaluated using the MTT assay (Nacalai Tesque, Kyoto, Japan). MDA-MB-231 and MCF-7 cells were seeded in 96-well plates at a density of 2 × 10^4^ cells/well and incubated overnight. After washing with PBS, the cells were treated with HA–His8 (0–1.0 mg/mL) in a phenol red-free medium for 12 h. The treatment medium was then replaced with fresh culture medium, and the cells were further incubated for 24 h. Subsequently, the MTT reagent was added, and the cells were incubated for 4 h at 37 °C. The resulting formazan crystals were dissolved in 2-propanol, and the absorbance was measured at 590 nm using a reference wavelength of 650 nm.

### 2.13. Statistical Analysis

Statistical analyses were performed using KaleidaGraph version 4.0 (Synergy Software, Reading, PA, USA). All quantitative data are presented as mean ± standard deviation (SD) from three independent experiments (*n* = 3), unless otherwise stated. Comparisons between two independent groups were performed using Welch’s unpaired *t*-test (two-tailed). Statistical significance was set at *p*-value < 0.05. Exact *p*-values are provided where appropriate. Significance levels are denoted as ns (not significant), * *p* < 0.05, ** *p* < 0.01, and *** *p* < 0.001.

## 3. Results and Discussion

### 3.1. Synthesis of HA–His8

**Scheme 1:**
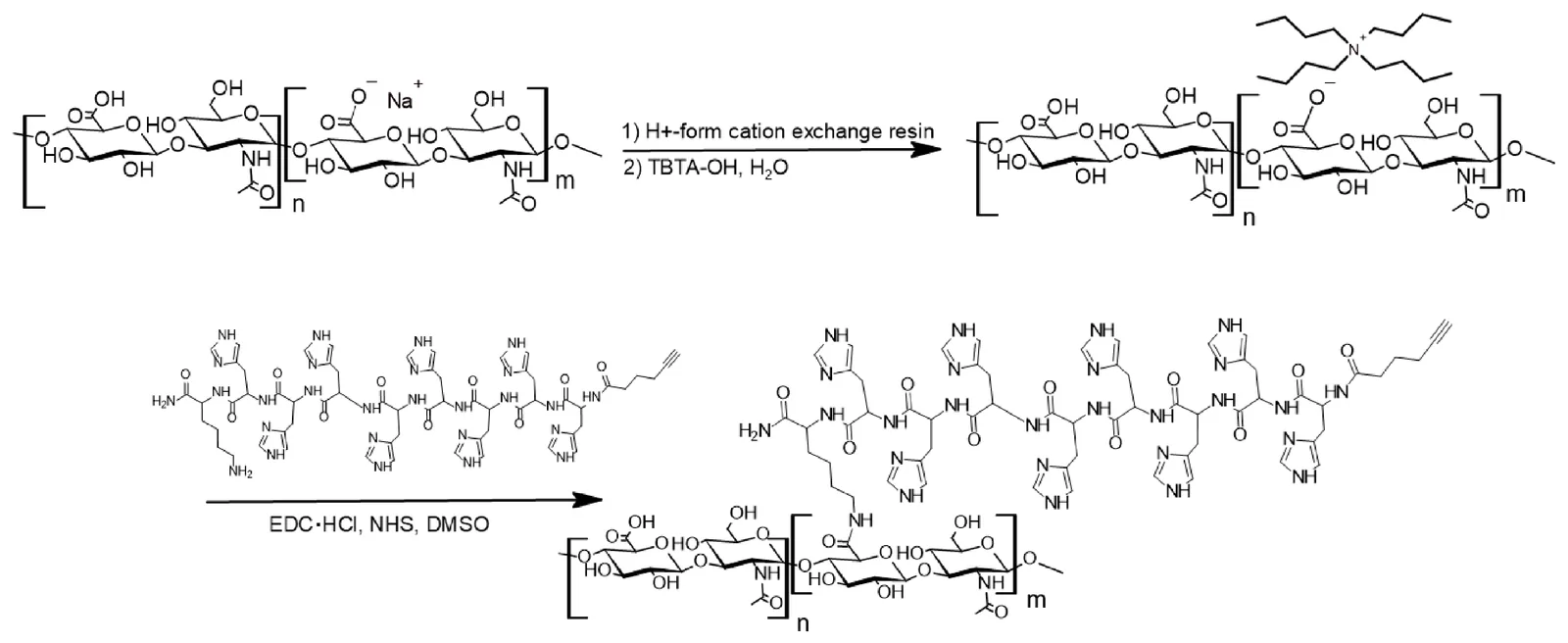
Synthetic route for hyaluronic acid (HA)–octahistidine (His8).

**Figure 2:**
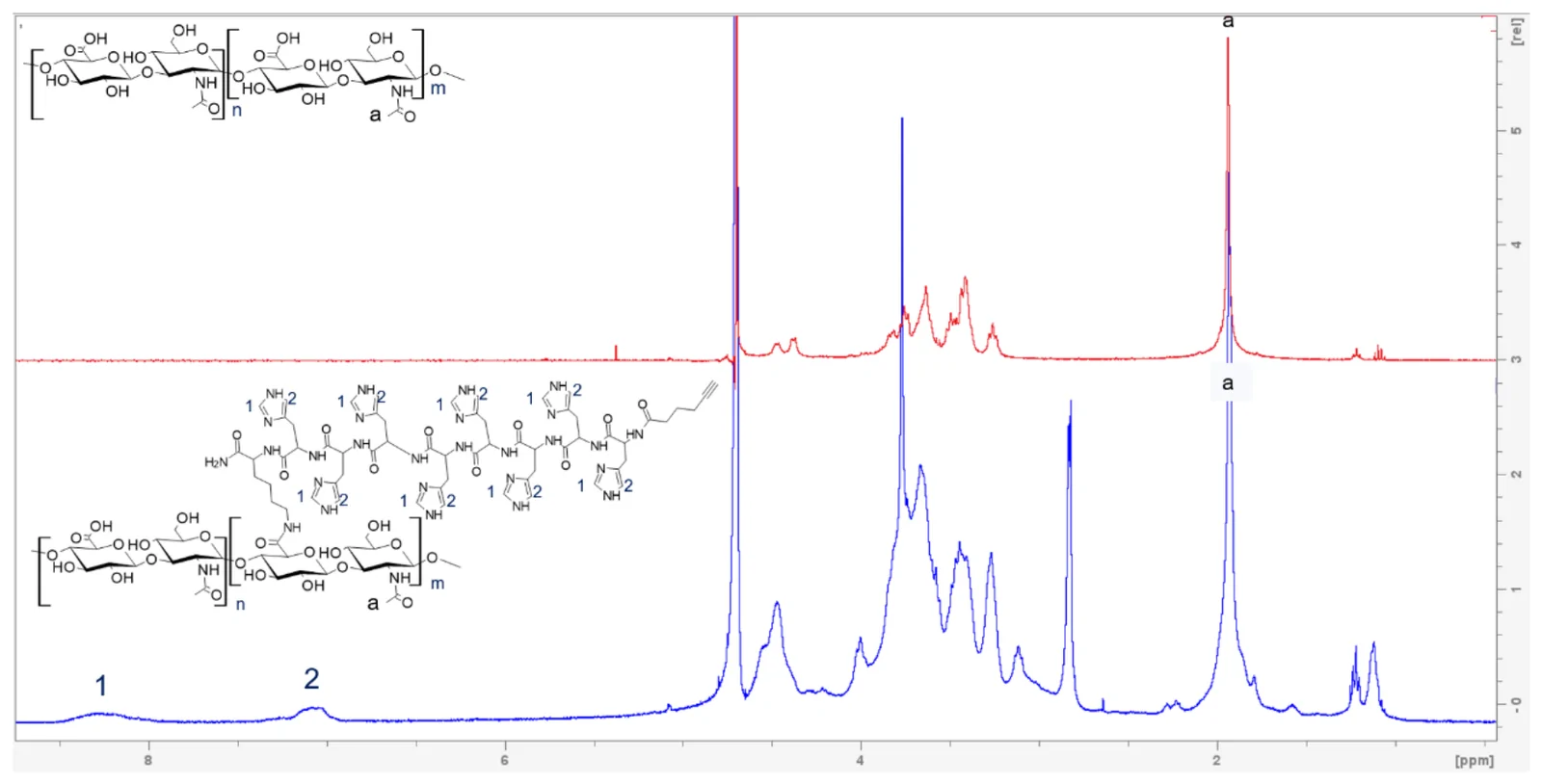
Comparison of the ^1^H NMR spectra of hyaluronic acid (HA) and hyaluronic acid–octahistidine (HA–His8).

HA–His8 was synthesized via amide bond formation using the EDC/NHS coupling chemistry (Scheme 1). Prior to conjugation with HA, the structure and molecular weight of the synthesized His8 were characterized by ^1^H NMR and MALDI-TOF MS. In the MALDI-TOF mass spectrum, the major ion peak was observed at m/z 1336.64, consistent with the calculated value for [M + H]+ (m/z 1336.43) (Figure S1). Furthermore, the ^1^H NMR spectrum showed characteristic signals attributable to the aromatic ring protons of His8, supporting the structure of the synthesized His8 (Figure S2). Successful conjugation of His8 to HA was confirmed by ^1^H NMR spectroscopy (Figure 2). In addition to the characteristic acetyl proton signal of HA (*δ* ≈ 2.0 ppm), characteristic signals corresponding to the imidazole ring protons of the histidine residues were observed at *δ* ≈ 7.1 and 7.8 ppm, confirming the successful introduction of His8 onto the HA backbone.

The degree of His8 substitution was determined from the integral ratio of the imidazole proton signal of histidine to the acetyl proton signal of HA, which was used as an internal reference. The degree of substitution was calculated to be 4.66 mol% relative to the carboxyl groups of HA.

In this study, His8 was introduced into HA to impart buffering capacity and endosomal escape capability, while preserving the CD44-targeting property of HA. The relatively low degree of substitution indicated that most of the carboxyl groups of HA remained unmodified, suggesting that the intrinsic physicochemical properties and CD44-binding characteristics of HA were largely preserved while introducing the functional properties of His8.

### 3.2. Physicochemical Characterization of HA–His8

**Figure 3:**
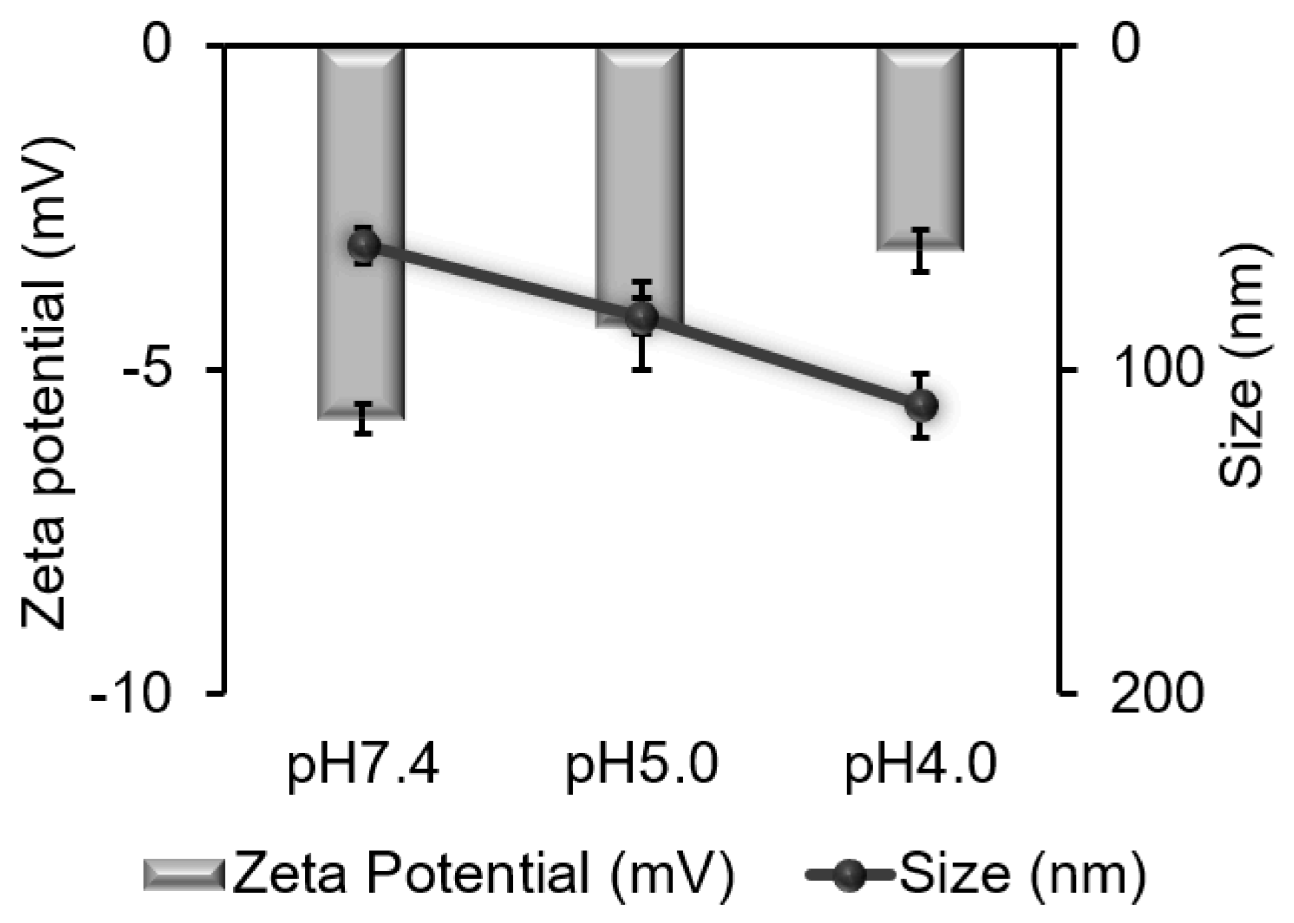
pH-dependent changes in the intensity-weighted hydrodynamic diameter and zeta potential of hyaluronic acid–octahistidine (HA–His8).

To investigate the pH-dependent changes in the physicochemical properties of HA–His8, the particle size and zeta potential were measured at pH 7.4, 5.0, and 4.0 (Figure 3 and Table 1). At pH 7.4, HA–His8 exhibited a particle size of 61.5 nm and a zeta potential of −5.75 mV. As the pH decreased, the particle size increased from 61.5 to 111.0 nm, while the zeta potential became less negative, changing from −5.75 to −3.16 mV. Furthermore, Cy–HA–His8, which was used for the subsequent cellular studies, showed a similar pH-dependent trend in hydrodynamic diameter and zeta potential to unlabeled HA–His8 (Figure S3 and Table S1), indicating that Cy5.5 labeling did not substantially alter the physicochemical behavior of HA–His8. To further characterize the DLS profiles and assess the potential contribution of large aggregates, intensity- and number-weighted size distributions of HA–His8 were also examined at each pH (Figure S4). The intensity-weighted distributions showed predominant populations in the nanoscale range without a distinct population at larger sizes, and the corresponding number-weighted distributions also showed predominant nanoscale populations. These distribution profiles support that the observed DLS results were not solely dominated by scattering from a small number of large aggregates.

The change in zeta potential is likely attributable to protonation of the imidazole groups of the histidine residues under acidic conditions, which partially neutralizes the negative charges derived from the carboxyl groups of HA. In addition, the increase in particle size suggests that protonation of histidine residues alters intermolecular interactions, resulting in changes in the apparent association state of HA–His8. These results indicate that the surface charge and hydrodynamic diameter of HA–His8 change with decreasing pH. Such pH-dependent physicochemical changes may influence the intracellular behavior of HA–His8 under the acidic conditions encountered in endosomes. Although the intensity- and number-weighted distributions provide additional information regarding the DLS profiles and the potential contribution of large aggregates, DLS measures hydrodynamic size in solution and does not directly provide morphological information. Therefore, the nanoscale hydrodynamic diameters observed in this study should not be interpreted as evidence for the formation of structurally defined nanoparticles, but rather as reflecting nanoscale hydrodynamic association states of HA–His8 in aqueous solution.

### 3.3. Evaluation of the Buffering Capacity of HA–His8

**Figure 4:**
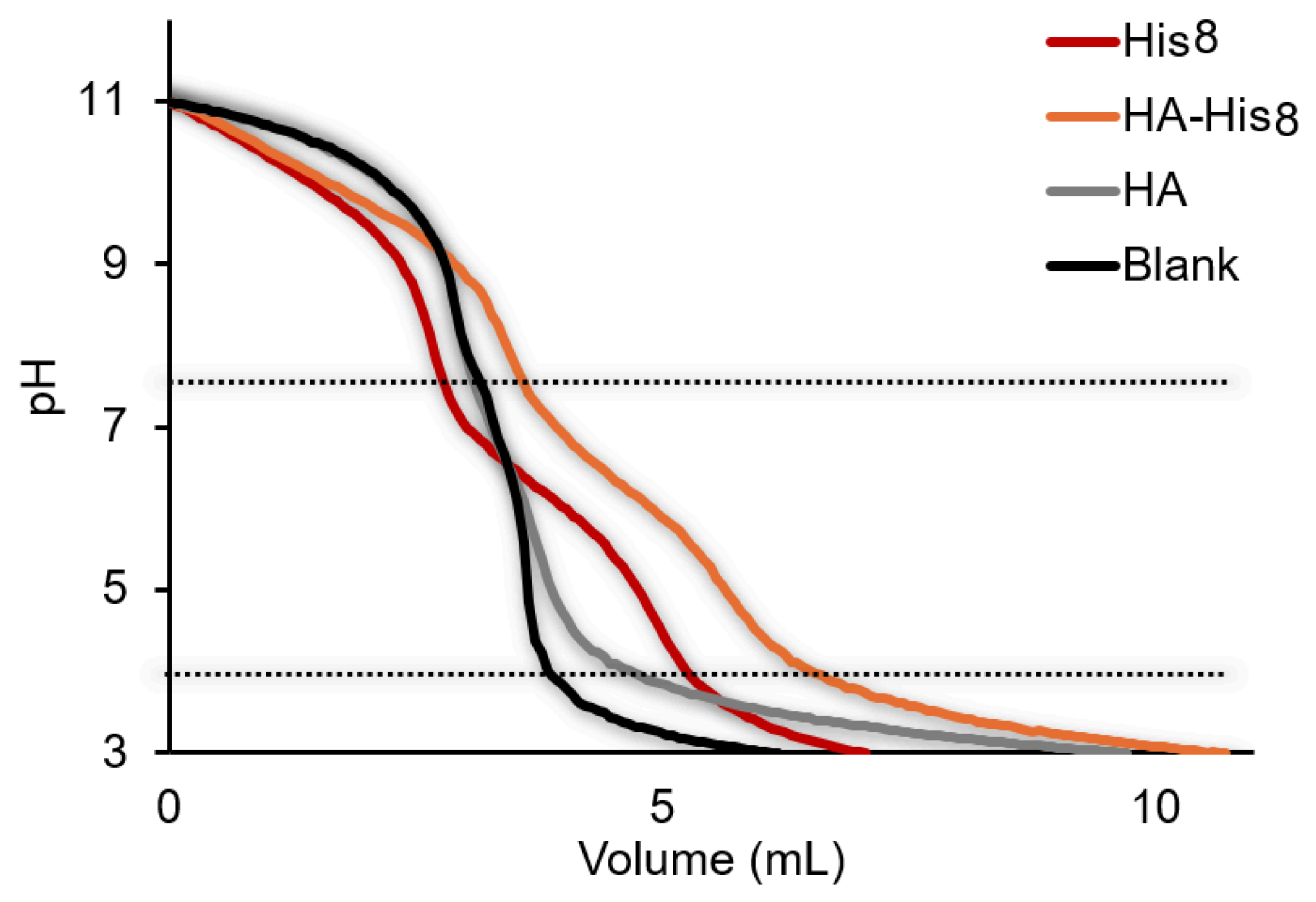
Acid–base titration curves of octahistidine (His8), hyaluronic acid–octahistidine (HA–His8), hyaluronic acid (HA), and the blank solution. Titration was performed using 0.01 M hydrochloric acid (HCl) to evaluate the buffering capacity of His8 and HA–His8 over the endosomal pH range (pH 7.4–4.0).

The buffering capacity of HA–His8 was evaluated by acid–base titration (Figure 4 and Table 2). The blank solution required 0.50 mL of 0.01 M HCl to decrease the pH from 7.4 to 4.0. In contrast, His8 and HA–His8 required 2.39 mL and 2.84 mL of HCl, respectively, indicating greater buffering capacity than the blank. Although HA exhibited a more gradual titration curve than the blank, its buffering capacity could not be calculated because it did not contain protonatable amino groups. The buffering capacities between pH 7.4 and 4.0 were calculated to be 78.42% for His8 and 97.13% for HA–His8, demonstrating that HA–His8 possessed a higher buffering capacity than free His8.

The imidazole group of histidine has a *pK_a_* of approximately 6.0 and is readily protonated under mildly acidic conditions in endosomes. Therefore, the enhanced buffering capacity of HA–His8 can be attributed to the efficient proton-accepting ability of the imidazole groups. Furthermore, the conjugation of His8 to the HA backbone may increase the local density of histidine residues within a single polymer chain, resulting in a greater buffering capacity than that of free His8.

These results demonstrate that HA–His8 possesses excellent proton-buffering ability under acidic conditions that mimic the endosomal environment. This physicochemical property is consistent with the intracellular localization results described below and supports the potential of HA–His8 to facilitate endosomal escape after cellular uptake.

### 3.4. Intracellular Localization and Endosomal Escape of HA–His8 in Cells with Different CD44 Expression Levels

To evaluate the intracellular localization and endosomal escape capability of HA–His8, the intracellular behaviors of Cy–HA–His8 and unmodified Cy–HA were investigated by confocal laser scanning microscopy (CLSM) using MDA-MB-231 and MCF-7 cells, which exhibited different levels of CD44 expression. We previously reported that MDA-MB-231 cells express substantially higher levels of CD44 than MCF-7 cells. Therefore, these cell lines were used as CD44-high and CD44-low cell models, respectively. Cy5.5-labeled polymers were prepared using Sulfo-Cyanine5.5 amine according to the same procedure as that used for HA–His8 (see Supporting Information for details).

**Figure 5:**
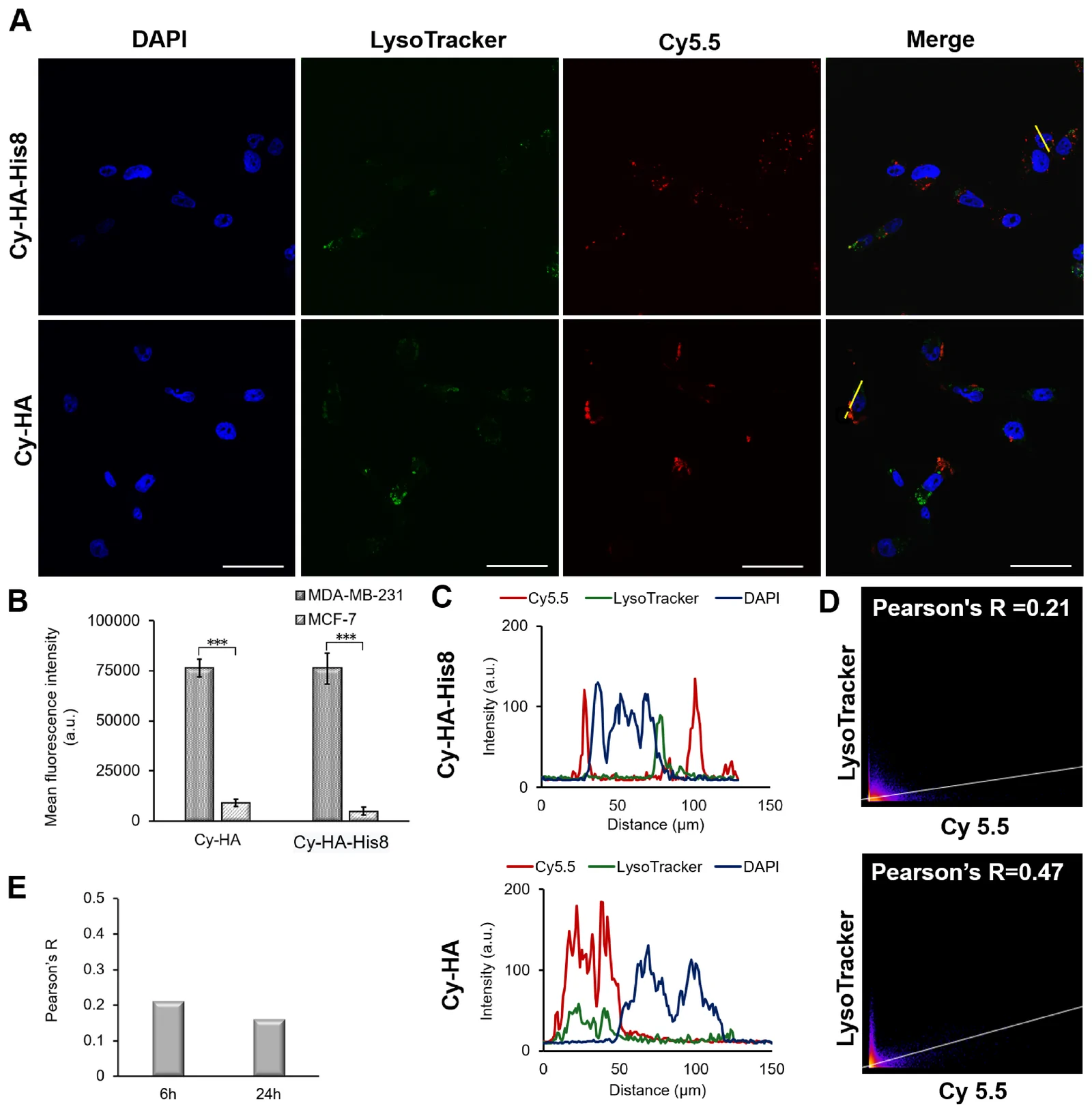
Intracellular localization and endosomal escape of Cy5.5-labeled HA–His8 (Cy–HA–His8) after 6 h incubation in cells with different CD44 expression levels. (**A**) Representative confocal laser scanning microscopy (CLSM) images of MDA-MB-231 cells incubated with Cy–HA–His8 or Cy5.5-labeled HA (Cy–HA). Yellow arrows indicate the positions at which the fluorescence intensity line profiles shown in (**C**) were obtained. DAPI: λ_ex_ = 405 nm, λ_em_ = 430–470 nm (nuclei, blue); LysoTracker (Alexa Fluor 488): λ_ex_ = 488 nm, λ_em_ = 500–540 nm (lysosomes, green); Cy5.5: λ_ex_ = 635 nm, λ_em_ = 660–750 nm (red). Scale bars = 50 µm. (**B**) Quantification of the intracellular Cy5.5 fluorescence intensity in MDA-MB-231 and MCF-7 cells after incubation with Cy–HA or Cy–HA–His8. Data are presented as the mean ± SD (n = 4). n.s., not significant; *** *p* < 0.001. (**C**) Representative fluorescence intensity line profiles of Cy5.5, LysoTracker, and DAPI signals. (**D**) Representative Pearson’s correlation plots between Cy5.5 and LysoTracker fluorescence signals in MDA-MB-231 cells treated with Cy–HA–His8 or Cy–HA. (**E**) Time-dependent changes in the Pearson’s correlation coefficients between Cy5.5 and LysoTracker fluorescence signals in MDA-MB-231 cells treated with Cy–HA–His8. The data shown are representative of a single experiment.

After 6 h of incubation, strong intracellular Cy5.5 fluorescence was observed for both Cy–HA–His8 and Cy–HA in MDA-MB-231 cells (Figure 5A). In contrast, only minimal intracellular fluorescence was detected for both polymers in CD44-low MCF-7 cells, indicating limited cellular uptake (Figure S5). Quantitative analysis of intracellular Cy5.5 fluorescence intensity further demonstrated that both Cy–HA and Cy–HA–His8 exhibited significantly higher fluorescence intensities in MDA-MB-231 cells than in MCF-7 cells (Figure 5B, *** *p* < 0.001). These results suggest that HA-mediated cellular uptake is associated with CD44 expression. Recent studies have reported various HA-based anticancer delivery systems that exploit the CD44-binding ability of HA. HA derivatives and HA-modified nanocarriers have been utilized for the preferential delivery of anticancer agents to CD44-overexpressing tumor cells [21,34,35]. These studies support the utility of HA as a targeting material for anticancer drug delivery. However, even after CD44-mediated internalization, trafficking of HA-based carriers through the endosomal/lysosomal pathway may remain a barrier to efficient cytosolic delivery. In this context, HA–His8 was designed to combine the preferential cellular uptake of HA in CD44-high cells with His8-mediated modulation of intracellular trafficking.

Comparison of the intracellular localization patterns in MDA-MB-231 cells revealed distinct distributions of the two polymers. Cy–HA–His8 exhibited finely dispersed punctate fluorescence throughout the cytoplasm, whereas Cy–HA showed a more clustered fluorescence pattern that largely overlapped with LysoTracker-positive acidic vesicles (Figure 5A). These observations suggest that the His8 modification reduces the retention of HA within acidic vesicles and alters its intracellular localization.

The colocalization of Cy5.5 fluorescence with LysoTracker was further evaluated by line profile analysis and Pearson’s correlation coefficients. In the line profile analysis, the fluorescence peaks of Cy5.5 and LysoTracker showed relatively little overlap in cells treated with Cy–HA–His8, whereas a substantial overlap was observed in cells treated with Cy–HA (Figure 5C). These results suggest reduced localization of Cy–HA–His8 within acidic vesicles.

Pearson’s correlation coefficients were calculated to quantitatively evaluate the degree of colocalization (Figure 5D). The Pearson’s correlation coefficient was 0.21 for Cy–HA–His8-treated cells, whereas a higher value of 0.47 was obtained for Cy–HA-treated cells. Furthermore, the Pearson’s correlation coefficient further decreased to 0.16 after 24 h of incubation with Cy–HA–His8 (Figure 5E and Figure S6). Collectively, these findings indicate that Cy–HA–His8 exhibited lower colocalization with acidic vesicles than unmodified HA, and that this tendency became more pronounced over time.

Because the imidazole groups of His8 become protonated under the weakly acidic conditions of endosomes, the enhanced buffering capacity of HA–His8 may influence the ionic and osmotic environments within the endosomes, thereby contributing to the observed changes in intracellular localization. However, the present colocalization analysis did not directly demonstrate membrane disruption or a proton sponge mechanism; rather, it provided indirect evidence supporting enhanced endosomal escape following His8 modification.

Various delivery systems have been developed to overcome the endosomal barrier, including lipid nanoparticles (LNPs), cationic polymers, membrane-fusogenic peptides, and pH-responsive materials. Among these systems, LNPs have achieved clinical application for nucleic acid delivery because of their high cargo encapsulation capacity and ability to protect nucleic acids from degradation; however, endosomal escape remains one of the major rate-limiting steps for efficient intracellular delivery. Recent reviews have reported that, in some nanoparticle systems, less than 5% of internalized cargo reaches the cytosol from the endolysosomal pathway. However, the methods and definitions used to quantify endosomal escape vary considerably among studies, limiting direct quantitative comparisons between different delivery systems [36,37].

Unlike cargo-loaded formulations such as LNPs, HA–His8 developed in the present study integrates preferential uptake by CD44-high cells through HA with buffering functionality of His8 in the endosomal pH range within a single polymeric material. However, because cargo-loading capacity, release kinetics, and direct endosomal escape efficiency were not evaluated in the present study, HA–His8 cannot be concluded to be superior to existing delivery systems. Rather than serving as a direct replacement for established DDSs such as LNPs, HA–His8 should therefore be regarded as a functional polymeric material that combines CD44 targeting with modulation of intracellular trafficking. Collectively, these results suggested that HA contributes to the preferential cellular uptake of HA–His8 by CD44-high cells, whereas His8 reduces its retention within acidic vesicles after internalization. Thus, HA–His8 is a promising platform for intracellular drug delivery, which requires efficient cytosolic transport.

The present study focused on establishing the fundamental physicochemical properties and intracellular behavior of HA–His8; therefore, the loading and release of a specific therapeutic cargo were not evaluated. For the future development of HA–His8 as a therapeutic delivery system, an appropriate cargo-loading strategy should be selected according to the molecular weight, charge, hydrophobicity, and intracellular site of action of the intended cargo. The loading capacity, release profile, intracellular delivery efficiency, and biological activity of cargo-loaded HA–His8 will therefore need to be systematically evaluated in subsequent studies. These investigations represent an important next step toward translating the fundamental material properties demonstrated here into a therapeutic DDS.

### 3.5. HA–His8 Promotes Endosomal/Lysosomal Leakage in MDA-MB-231 Cells

**Figure 6:**
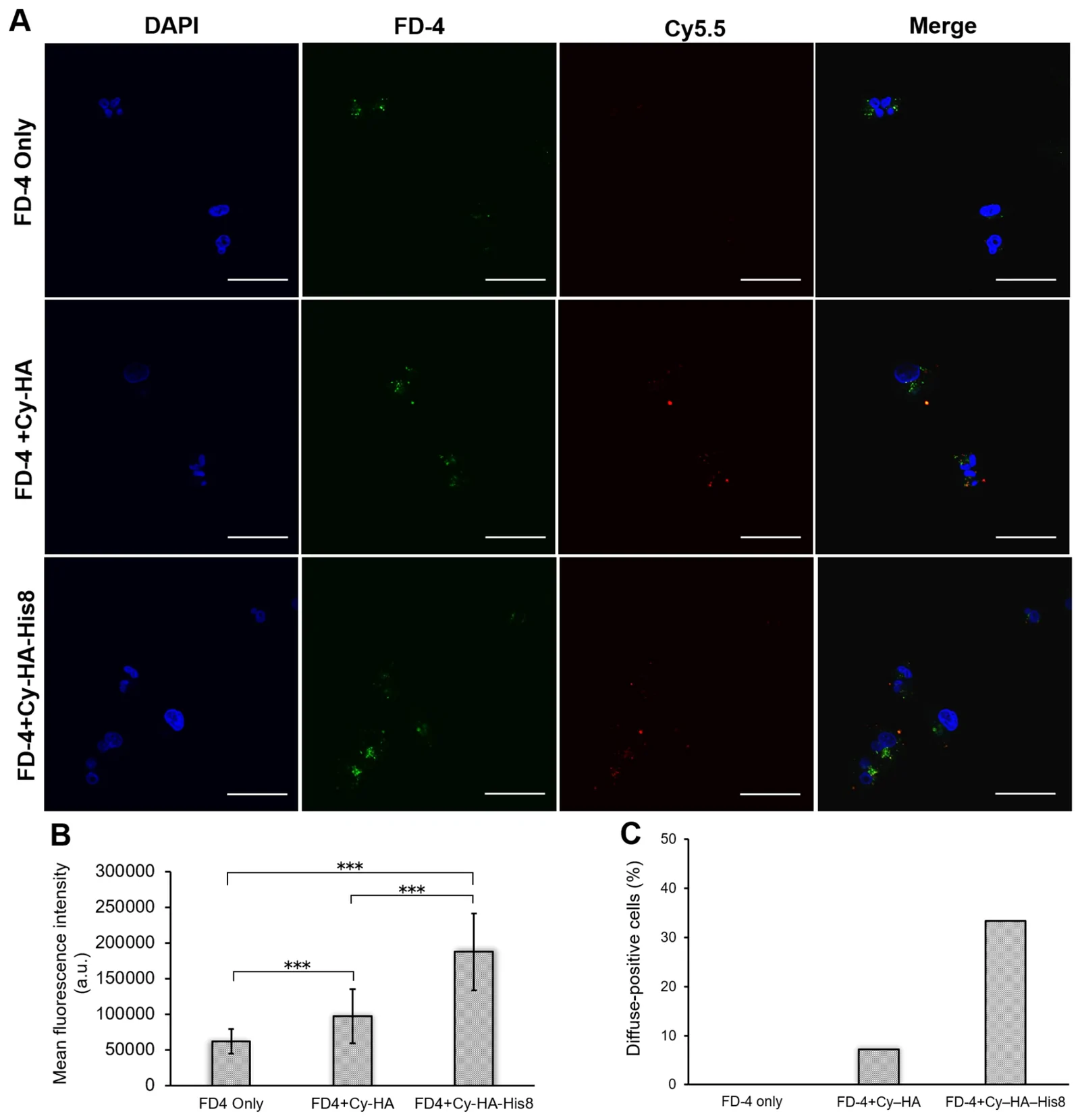
Evaluation of endosomal/lysosomal leakage using 4-kDa FITC–dextran (FD-4) at 6 h. MDA-MB-231 cells were incubated with FD-4 alone or in the presence of Cy–HA or Cy–HA–His8. (**A**) Representative confocal fluorescence microscopy images of intracellular FD-4 distribution. Blue, DAPI: λ_ex_ = 405 nm, λ_em_ = 430–470 nm (nuclei, blue); FD-4 (Alexa Fluor 488): λ_ex_ = 488 nm, λ_em_ = 500–540 nm (lysosomes, green); Cy5.5: λ_ex_ = 635 nm, λ_em_ = 660–750 nm (red). Scale bars = 50 μm. (**B**) Intracellular FD-4 fluorescence intensity per cell. Data are presented as mean ± SD. *** *p* < 0.001. (**C**) Percentage of cells exhibiting diffuse cytoplasmic FD-4 fluorescence. Cells were classified as punctate or diffuse based on the intracellular FD-4 distribution. Cells pooled from three microscopic fields were analyzed for each group.

Colocalization analysis using LysoTracker Green showed decreased colocalization of Cy–HA–His8 with LysoTracker compared with Cy–HA after 6 h of incubation, suggesting that His8 modification altered the intracellular localization of HA. However, because LysoTracker preferentially accumulates in acidic intracellular compartments, reduced colocalization alone cannot distinguish escape from these compartments from changes in their acidic environment. Therefore, to further evaluate leakage from intracellular vesicular compartments, we examined the intracellular distribution of membrane-impermeable 4-kDa FITC–dextran (FD4) at the same 6-h time point used for the LysoTracker analysis (Figure 6).

In the FD4-only and Cy–HA-treated groups, FD4 fluorescence was observed predominantly as discrete intracellular puncta. In contrast, cells treated with Cy–HA–His8 exhibited not only punctate fluorescence but also a more diffuse intracellular distribution of FD4 fluorescence (Figure 6A). Moreover, the FD4 fluorescence intensity per cell was significantly higher in the Cy–HA–His8-treated group than in the Cy–HA-treated group (Figure 6B). Classification of the intracellular FD4 distribution as punctate or diffuse further showed a higher percentage of cells exhibiting a diffuse FD4 fluorescence pattern in the Cy–HA–His8-treated group (Figure 6C).

Unlike LysoTracker, FD4 does not rely on preferential accumulation in acidic intracellular compartments for its localization. Therefore, the change from a predominantly punctate FD4 pattern to a more diffuse intracellular distribution in the presence of Cy–HA–His8 is consistent with leakage of vesicularly entrapped FD4 from intracellular compartments. The increased FD4 fluorescence intensity per cell may also reflect differences in cellular uptake and was therefore not interpreted by itself as evidence of vesicular leakage. Rather, the altered spatial distribution of FD4 provides additional evidence, independent of LysoTracker colocalization, supporting an effect of His8 modification on intracellular vesicular retention. Nevertheless, the FD4 assay does not directly visualize membrane disruption or establish the underlying mechanism. Taken together with the reduced LysoTracker colocalization observed at the same time point, these findings support the possibility that His8 modification facilitates endosomal/lysosomal leakage and alters the intracellular trafficking of HA.

### 3.6. Evaluation of Cytotoxicity of HA–His8

**Figure 7:**
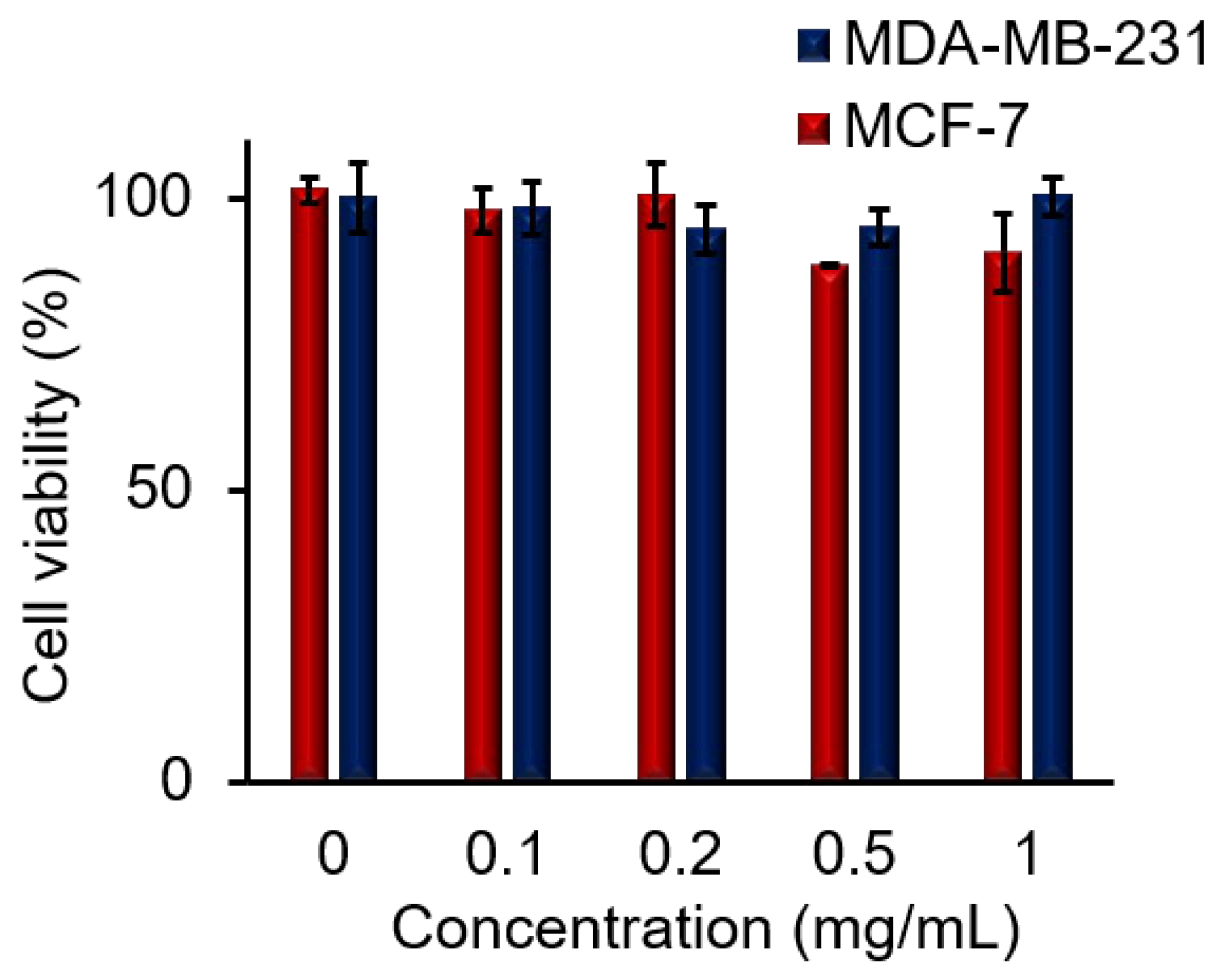
Cytotoxicity of HA–His8 in MDA-MB-231 and MCF-7 cells. Cell viability was evaluated by using the MTT assay following incubation with hyaluronic acid–octahistidine (HA–His8) at the indicated concentrations. Data are presented as the mean ± SD (n = 3). SD, standard deviation.

The cytotoxicity of HA–His8 was evaluated in MDA-MB-231 and MCF-7 cells via the MTT assay (Figure 7). HA–His8 maintained cell viability above 90% in both cell lines at concentrations of up to 0.2 mg/mL. At 0.5 mg/mL, a slight decrease in cell viability was observed in the MDA-MB-231 cells, whereas the MCF-7 cells maintained approximately 90% viability. Even at the highest tested concentration (1.0 mg/mL), both cell lines retained cell viability of approximately 85%, indicating no marked cytotoxicity.

These results demonstrate that HA–His8 exhibits high biocompatibility across the tested concentration range. Importantly, the concentration used for the intracellular localization and endosomal escape studies (0.5 mg/mL) maintained high cell viability in both cell lines, indicating that the observed differences in intracellular distribution and lysosomal co-localization are unlikely to be attributable to cytotoxic effects. Collectively, these findings suggest that His8 modification enhances intracellular trafficking while preserving the favorable biocompatibility of HA, supporting the potential of HA–His8 as a platform material for intracellular delivery.

## 4. Conclusions

In this study, we developed a novel functional polymer, HA–His8, by introducing His8 into HA, which possesses CD44-targeting capability. Successful conjugation of His8 was confirmed by ^1^H NMR spectroscopy, and the degree of substitution was determined to be 4.66 mol% relative to the carboxyl groups of HA. HA–His8 exhibited a nanoscale hydrodynamic diameter under physiological conditions, which increased under acidic conditions together with changes in zeta potential. Furthermore, HA–His8 exhibited a higher buffering capacity than free His8, indicating its superior proton-accepting ability under acidic endosomal conditions.

Cellular studies demonstrated that HA–His8 was selectively internalized by CD44-high MDA-MB-231 cells, whereas negligible uptake was observed in CD44-low MCF-7 cells, indicating that HA-mediated cellular uptake is dependent on CD44 expression. Moreover, HA–His8 demonstrated lower colocalization with LysoTracker than unmodified HA, and colocalization further decreased over time, supporting reduced retention within acidic vesicular compartments following His8 modification. In addition, HA–His8 exhibited low cytotoxicity over the tested concentration range, indicating favorable biocompatibility.

Collectively, these findings suggest that His8 modification is a promising molecular design strategy for promoting intracellular trafficking beyond acidic vesicular compartments while retaining the preferential uptake characteristics of HA in CD44-high cells. HA–His8 therefore represents a potential functional polymeric platform for therapeutic cargos requiring efficient intracellular delivery.

## Figures and Tables

**Table 1:** Physicochemical properties of hyaluronic acid–octahistidine (HA–His8) at different pH levels. The hydrodynamic diameter represents the intensity-weighted mean diameter determined by DLS. DLS, dynamic light scattering; PDI, polydispersity index; SD, standard deviation.

pH	Zeta Potential ± SD (mV)	Size ± SD (nm)	PDI ± SD
7.4	−5.75 ± 0.237	61.5 ± 5.6	0.383 ± 0.063
5.0	−4.32 ± 0.670	83.5 ± 5.6	0.318 ± 0.109
4.0	−3.16 ± 0.322	111.0 ± 10.1	0.268 ± 0.165

**Table 2:** Buffering capacities of His8 and HA–His8 determined by acid–base titration. HCl, hydrochloric acid.

	0.01 M HCl (mL)	Buffering Capacity (%)
His8	2.39	78.42
HA–His8	2.84	97.13
Blank	0.50	n.d.

## Data Availability

The original contributions of this study are included in the article and Supplementary Materials. Further inquiries can be directed to the corresponding authors.

## Supplementary Materials

The following supporting information can be downloaded at: https://www.mdpi.com/article/10.3390/pharmaceutics18091137/s1, Supplementary Methods: Synthesis of Cy–HA; Synthesis of Cy5.5-labeled HA–His8 (Cy–HA–His8); Supplementary Figures: Figure S1: MALDI-TOF mass spectrum of His8; Figure S2: ^1^H NMR spectrum of His8 in D_2_O (400 MHz); Figure S3: pH-dependent changes in the hydrodynamic diameter and zeta potential of Cy–HA–His8; Figure S4: Intensity- and number-weighted size distributions of HA–His8 at different pH values; Figure S5: Representative CLSM images of CD44-low MCF-7 cells incubated with Cy–HA–His8 or Cy–HA for 6 h; Figure S6: Intracellular localization and endosomal escape evaluation of Cy–HA–His8 in cells with different CD44 expression levels after 24 h incubation. Supplementary Table: Table S1: Physicochemical properties of Cy–HA–His8 at different pH levels.

## References

[1] Walsh G., Walsh E. (2022). Biopharmaceutical Benchmarks 2022. Nat. Biotechnol..

[2] McGonigle P. (2025). How Biologics Have Changed the Drug Discovery Landscape. Annu. Rev. Pharmacol. Toxicol..

[3] Kumar S., Garg N.K., Jain A., Pandey P., Khopade A., Sawant K.K. (2024). Recent Progress in Macromolecules: From Current Therapeutic Strategies to Theranostic Applications. J. Drug Deliv. Sci. Technol..

[4] Fine J., Meksiriporn B., Tan J., Spangler J.B. (2024). Mechanism-Driven Design of Multispecific Antibodies for Targeted Disease Treatment. Annu. Rev. Chem. Biomol. Eng..

[5] Chen P., Cabral H. (2024). Enhancing Targeted Drug Delivery through Cell-Specific Endosomal Escape. ChemMedChem.

[6] Mai L.D., Wimberley S.C., Champion J.A. (2024). Intracellular Delivery Strategies Using Membrane-Interacting Peptides and Proteins. Nanoscale.

[7] Batistatou N., Kritzer J.A. (2024). Recent Advances in Methods for Quantifying the Cell Penetration of Macromolecules. Curr. Opin. Chem. Biol..

[8] Patel S., Kim J., Herrera M., Mukherjee A., Kabanov A.V., Sahay G. (2019). Brief Update on Endocytosis of Nanomedicines. Adv. Drug Deliv. Rev..

[9] Wang X., Qiu Y., Wang M., Zhang C., Zhang T., Zhou H., Zhao W., Zhao W., Xia G., Shao R. (2020). Endocytosis and Organelle Targeting of Nanomedicines in Cancer Therapy. Int. J. Nanomed..

[10] Chahal G., Helbig K., Parton R., Monson E. (2026). The Biology of Endosomal Escape: Strategies for Enhanced Delivery of Therapeutics. ACS Nano.

[11] Nagaraj H., Lehot V., Nasim N., Cicek Y.A., Goswami R., Jeon T., Rotello V.M. (2025). Breaking the Cellular Delivery Bottleneck: Recent Developments in Direct Cytosolic Delivery of Biologics. RSC Pharm..

[12] Varkouhi A.K., Scholte M., Storm G., Haisma H.J. (2011). Endosomal Escape Pathways for Delivery of Biologicals. J. Control. Release.

[13] Nakase I., Kobayashi S., Futaki S. (2010). Endosome-disruptive Peptides for Improving Cytosolic Delivery of Bioactive Macromolecules. Pept. Sci..

[14] Wang S. (2021). pH-Responsive Amphiphilic Carboxylate Polymers: Design and Potential for Endosomal Escape. Front. Chem..

[15] Lāce I., Cotroneo E.R., Hesselbarth N., Simeth N.A. (2023). Artificial Peptides to Induce Membrane Denaturation and Disruption and Modulate Membrane Composition and Fusion. J. Pept. Sci..

[16] Ferrer-Miralles N., Corchero J.L., Kumar P., Cedano J.A., Gupta K.C., Villaverde A., Vazquez E. (2011). Biological Activities of Histidine-Rich Peptides; Merging Biotechnology and Nanomedicine. Microb. Cell Factories.

[17] Pichon C., Roufaï M.B., Monsigny M., Midoux P. (2000). Histidylated Oligolysines Increase the Transmembrane Passage and the Biological Activity of Antisense Oligonucleotides. Nucleic Acids Res..

[18] Pack D.W., Putnam D., Langer R. (2000). Design of Imidazole-Containing Endosomolytic Biopolymers for Gene Delivery. Biotechnol. Bioeng..

[19] Moreira C., Oliveira H., Pires L.R., Simões S., Barbosa M.A., Pêgo A.P. (2009). Improving Chitosan-Mediated Gene Transfer by the Introduction of Intracellular Buffering Moieties into the Chitosan Backbone. Acta Biomater..

[20] He J., Xu S., Mixson A.J. (2020). The Multifaceted Histidine-Based Carriers for Nucleic Acid Delivery: Advances and Challenges. Pharmaceutics.

[21] Yukimura T., Seki T., Seki T. (2026). Dual-Trigger Hyaluronic Acid Nanoprodrug Incorporating a 2-Nitrobenzenesulfonyl Linker for CD44-Targeted and Glutathione-Responsive Drug Delivery. Int. J. Pharm..

[22] Huang G., Huang H. (2018). Application of Hyaluronic Acid as Carriers in Drug Delivery. Drug Deliv..

[23] Tripodo G., Trapani A., Torre M.L., Giammona G., Trapani G., Mandracchia D. (2015). Hyaluronic Acid and Its Derivatives in Drug Delivery and Imaging: Recent Advances and Challenges. Eur. J. Pharm. Biopharm..

[24] Toole B.P. (2009). Hyaluronan-CD44 Interactions in Cancer: Paradoxes and Possibilities. Clin. Cancer Res..

[25] Choi K.Y., Min K.H., Na J.H., Choi K., Kim K., Park J.H., Kwon I.C., Jeong S.Y. (2009). Self-Assembled Hyaluronic Acid Nanoparticles as a Potential Drug Carrier for Cancer Therapy: Synthesis, Characterization, and In Vivo Biodistribution. J. Mater. Chem..

[26] Prestwich G.D. (2011). Hyaluronic Acid-Based Clinical Biomaterials Derived for Cell and Molecule Delivery in Regenerative Medicine. J. Control. Release.

[27] Fu C.-P., Cai X.-Y., Chen S.-L., Yu H.-W., Fang Y., Feng X.-C., Zhang L.-M., Li C.-Y. (2023). Hyaluronic Acid-Based Nanocarriers for Anticancer Drug Delivery. Polymers.

[28] Takeda M., Terasawa H., Sakakura M., Yamaguchi Y., Kajiwara M., Kawashima H., Miyasaka M., Shimada I. (2003). Hyaluronan Recognition Mode of CD44 Revealed by Cross-Saturation and Chemical Shift Perturbation Experiments. J. Biol. Chem..

[29] Kwon M.Y., Wang C., Galarraga J.H., Puré E., Han L., Burdick J.A. (2019). Influence of Hyaluronic Acid Modification on CD44 Binding towards the Design of Hydrogel Biomaterials. Biomaterials.

[30] Choi K.Y., Saravanakumar G., Park J.H., Park K. (2012). Hyaluronic Acid-Based Nanocarriers for Intracellular Targeting: Interfacial Interactions with Proteins in Cancer. Colloids Surf. B Biointerfaces.

[31] Kanai R., Seki T., Yukimura T., Komatsu S., Itakura S., Arce F.J., See G.L., Kiba Y., Kitamura M., Kodama N. (2026). Potential Enhancement of Topical Drug Delivery Using Grapefruit-Derived Nanoparticles Modified Using TAT Peptide. Pharm. Res..

[32] Hirakura T., Yasugi K., Nemoto T., Sato M., Shimoboji T., Aso Y., Morimoto N., Akiyoshi K. (2010). Hybrid Hyaluronan Hydrogel Encapsulating Nanogel as a Protein Nanocarrier: New System for Sustained Delivery of Protein with a Chaperone-like Function. J. Control. Release.

[33] D’Este M., Eglin D., Alini M. (2014). A Systematic Analysis of DMTMM vs EDC/NHS for Ligation of Amines to Hyaluronan in Water. Carbohydr. Polym..

[34] Wu Y., Li J., Liu L., Chu X., Zhong M., Li H., Zhao C., Fu H., Sun Y., Li Y. (2024). Hyaluronic Acid Nanoparticles for Targeted Oral Delivery of Doxorubicin: Lymphatic Transport and CD44 Engagement. Int. J. Biol. Macromol..

[35] Mugundhan S.L., Mohan M. (2025). Hyaluronic Acid-Coated Capecitabine Nanostructures for CD44 Receptor-Mediated Targeting in Breast Cancer Therapy. RSC Adv..

[36] Henao M.C., Ocasion C., Puentes P.R., González-Melo C., Quezada V., Cifuentes J., Yepes A., Burgos J.C., Cruz J.C., Reyes L.H. (2022). Translocating Peptides of Biomedical Interest Obtained from the Spike (S) Glycoprotein of the SARS-CoV-2. Membranes.

[37] Torres-Vanegas J.D., Cifuentes J., Puentes P.R., Quezada V., Garcia-Brand A.J., Cruz J.C., Reyes L.H. (2022). Assessing Cellular Internalization and Endosomal Escape Abilities of Novel BUFII-Graphene Oxide Nanobioconjugates. Front. Chem..

