# Supplementary material for: Evaluation of Histidine–Octamer-Modified Hyaluronic Acid as a Cytosolic Drug Delivery Material via CD44-Mediated Cellular Uptake and Endosomal Escape
Source: Pharmaceutics. 2026 Sep 10;18(9):1137. doi: 10.3390/pharmaceutics18091137 (PMC13610421; doi:10.3390/pharmaceutics18091137)
Supplement: Supplementary file 1 [file pharmaceutics-18-01137-s001.zip › pharmaceutics-4507900-supplementary.pdf]

Supplementary Methods: Synthesis of Cy–HA

HA–TBA (90 mg, 2.0 μmol; 224 μmol of carboxyl groups) was dissolved in distilled water (9.0 mL). Cy5.5 (4.83 mg, 22.4 μmol) dissolved in DMF (6.0 mL) was added to the HA solution. EDC·HCl (85.89 mg, 896 μmol) and NHS (51.56 mg, 896 μmol) were subsequently added, and the reaction mixture was stirred at room temperature for 24 h. The reaction mixture was dialyzed against excess distilled water for 24 h, during which the external water was replaced 2–3 times. The purified solution was lyophilized to obtain Cy–HA as a solid[1].

Synthesis of Cy5.5-labeled HA–His8 (Cy–HA–His8)

Cy–HA (49.2 mg, 0.5 μmol; corresponding to 56 μmol of carboxyl groups) was dissolved in anhydrous DMSO (3.5 mL). EDC·HCl (42.94 mg, 224 μmol) and NHS (25.76 mg, 224 μmol) were added, and the reaction mixture was stirred for 1 h at room temperature to activate the carboxyl groups.

His8 (37.4 mg, 28 μmol), dissolved in anhydrous DMSO (2.5 mL), was added dropwise to the activated Cy–HA solution, followed by DIPEA (52.4 μL, 308 μmol). The reaction mixture was stirred at room temperature for 24 h.

The reaction mixture was dialyzed against excess distilled water for 24 h, during which the external water was replaced 2–3 times. The product was then dissolved in 5% NaCl solution at an HA concentration of 10 mg/mL and further dialyzed against distilled water for 24 h using regenerated cellulose dialysis tubing (MWCO 15 kDa). The purified solution was lyophilized to obtain Cy–HA–His8 as a powder.

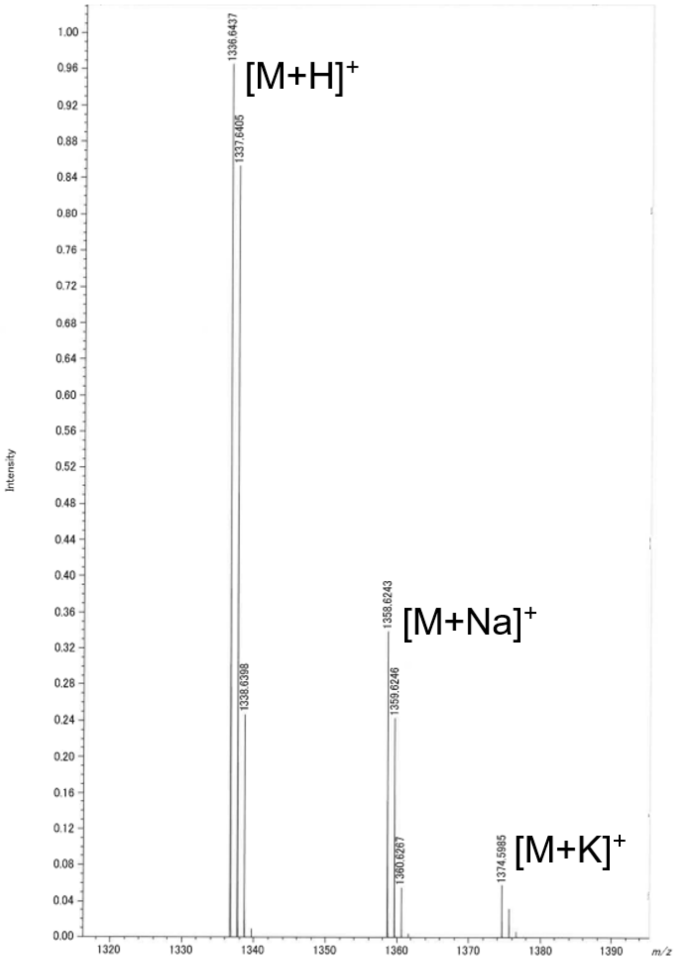

Figure S1. MALDI-TOF mass spectrum of His8. The observed signals were assigned to $[M + H]^+$, $[M + Na]^+$, and $[M + K]^+$ ions.

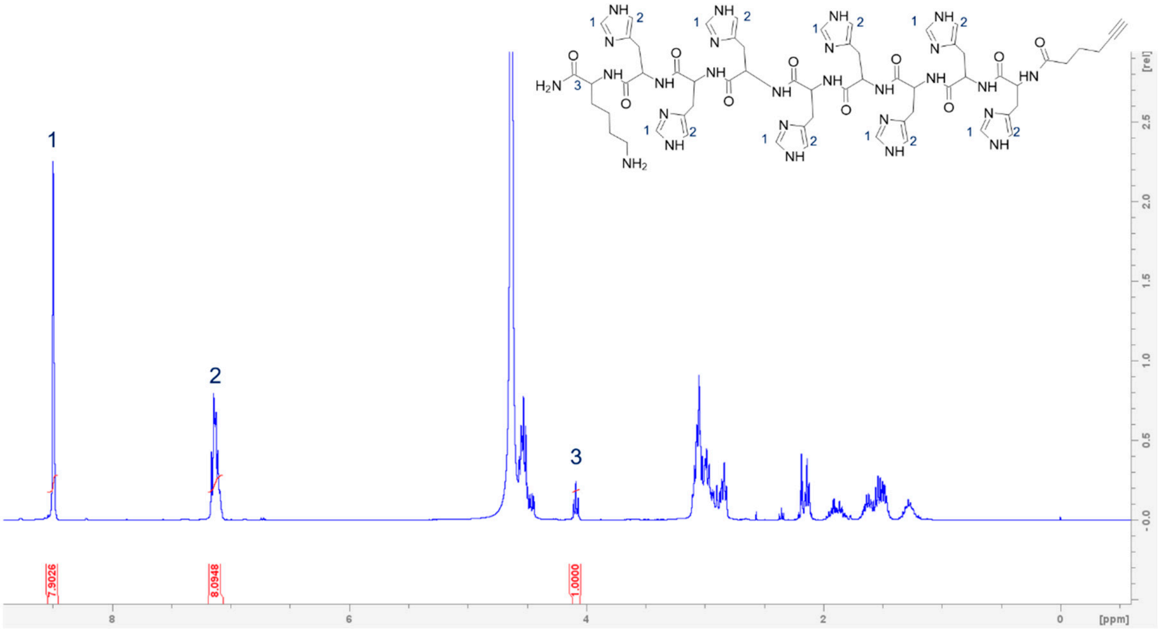

Figure S2. ^1H NMR spectrum of His8 in D_2O (400 MHz).

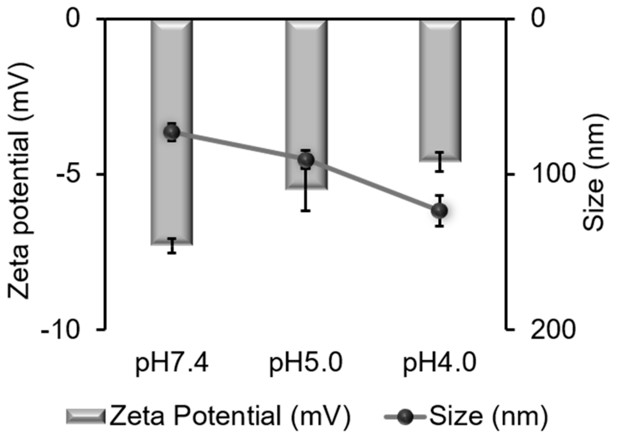

Figure S3. pH-dependent changes in the hydrodynamic diameter and zeta potential of Cy–HA–His8.

Table S1. Physicochemical properties of Cy–HA–His8 at different pH levels.

pH	Zeta Potential (mV)±SD	Size (nm) ±SD	PDI±SD
7.4	-7.31±0.715	72.9±6.5	0.313±0.053
5.0	-5.52±0.491	90.4±4.7	0.348±0.112
4.0	-4.31±0.538	123.4±12.2	0.299±0.165

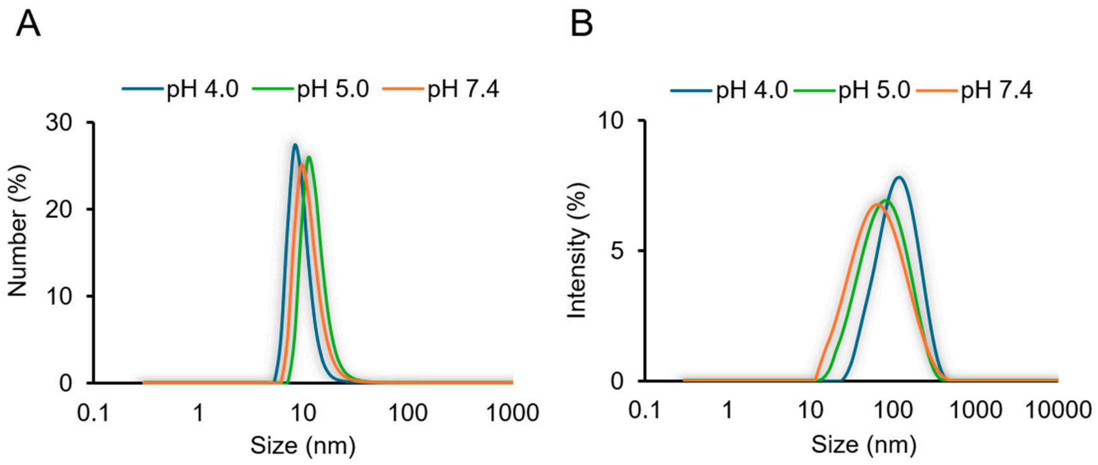

Figure S4. Intensity- and number-weighted size distributions of HA–His8 at different pH values. (A) Intensity-weighted and (B) number-weighted size distributions of HA–His8 at pH 4.0, 5.0, and 7.4, as determined by DLS.

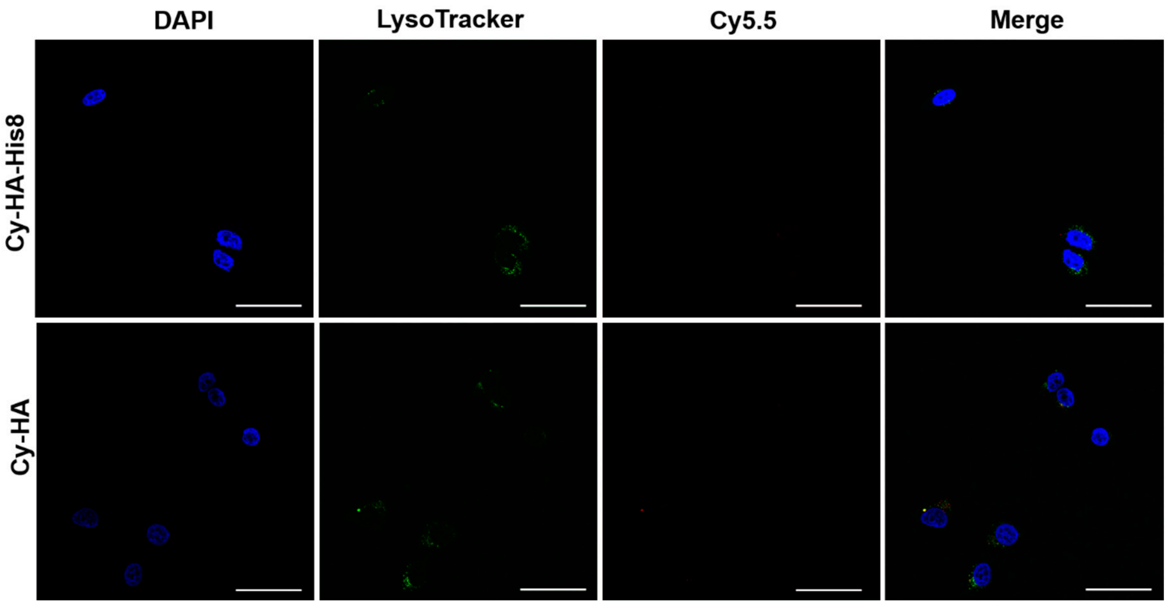

Figure S5. Representative CLSM images of CD44-low MCF-7 cells incubated with Cy–HA–His8 or Cy–HA for 6 h. DAPI λ_{ex}=405 nm, λ_{em}=430–470 nm (Nuclear-blue), Alexa Fluor 488 λ_{ex}=488 nm, λ_{em}=500–540 nm (Lysosome-green), Cy5.5 λ_{ex} = 635 nm, λ_{em} = 660–750 nm (Cy5.5—red), 50 μm scale.

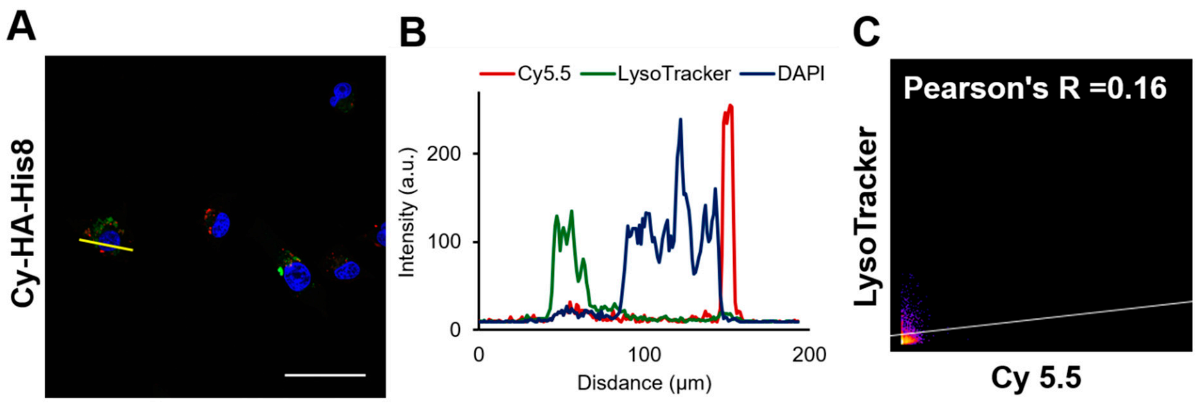

Figure S6. Intracellular localization and endosomal escape evaluation of Cy–HA–His8 in cells with different CD44 expression levels after 24 h incubation. (A) Representative CLSM images of MDA-MB-231 cells incubated with Cy–HA–His8. Yellow lines indicate the positions at which the fluorescence intensity line profiles shown in (B) were obtained. (B) Representative fluorescence intensity line profiles of Cy5.5, LysoTracker, and DAPI signals. (C) Representative Pearson's correlation plot between Cy5.5 and LysoTracker fluorescence signals in MDA-MB-231 cells treated with Cy–HA–His8. DAPI: λ_{ex} = 405 nm, λ_{em} = 430–470 nm (nuclei, blue); LysoTracker (Alexa Fluor 488): λ_{ex} = 488 nm, λ_{em} = 500–540 nm (lysosomes, green); Cy5.5: λ_{ex} = 635 nm, λ_{em} = 660–750 nm (red). Scale bar = 50 μm.

Reference

1. Lee, Y.; Sugihara, K.; Gillilland, M.G.; Jon, S.; Kamada, N.; Moon, J.J. Hyaluronic Acid–Bilirubin Nanomedicine for Targeted Modulation of Dysregulated Intestinal Barrier, Microbiome and Immune Responses in Colitis. *Nat. Mater.* **2020**, *19*, 118–126, doi:10.1038/s41563-019-0462-9.
